# Predictive Assessment of Quantitative Ultra-Widefield Angiographic Features for Future Need for Anti-VEGF Therapy in Diabetic Eye Disease

**DOI:** 10.3390/jpm12040608

**Published:** 2022-04-10

**Authors:** Alice C. Jiang, Duriye Damla Sevgi, Christopher Mugnaini, Jon Whitney, Sunil K. Srivastava, Katherine E. Talcott, Ming Hu, Jamie L. Reese, Justis P. Ehlers

**Affiliations:** 1 The Tony and Leona Campane Center for Excellence in Image-Guided Surgery and Advanced Imaging Research, Cole Eye Institute, Cleveland Clinic, Cleveland, OH 44195, USA; acj43@case.edu (A.C.J.); ddamlasevgi@gmail.com (D.D.S.); mugnaic@ccf.org (C.M.); whitnej@ccf.org (J.W.); srivass2@ccf.org (S.K.S.); talcotk@ccf.org (K.E.T.); hum@ccf.org (M.H.); reesej3@ccf.org (J.L.R.); 2 School of Medicine, Case Western Reserve University, Cleveland, OH 44195, USA; 3 Vitreoretinal Service, Cole Eye Institute, Cleveland Clinic, Cleveland, OH 44195, USA; 4 Department of Quantitative Health Sciences, Cleveland Clinic, Cleveland, OH 44195, USA

**Keywords:** anti-VEGF, diabetic retinopathy, personalized treatment, predicting anti-VEGF, fluorescein angiography, quantitative image analysis, artificial intelligence, predictive modeling

## Abstract

The objective of this study was to identify biomarkers that predict a future need for anti-VEGF therapy in diabetic retinopathy (DR). Eyes with DR that underwent ultra-widefield angiography (UWFA) and had at least a 1 year follow-up were grouped based on future anti-VEGF treatment requirements: (1) not requiring treatment, (2) immediate treatment (within 3 months of UWFA), and (3) delayed treatment (after 3 months of UWFA). Quantitative UWFA features and clinical factors were evaluated. Random forest models were built to differentiate eyes requiring immediate and delayed treatment from the eyes not requiring treatment. A total of 173 eyes were included. The mean follow-up was 22 (range: 11–43) months. The macular leakage index, panretinal leakage index, presence of DME, and visual acuity were significantly different in eyes requiring immediate (*n* = 38) and delayed (*n* = 34) treatment compared to eyes not requiring treatment (*n* = 101). Random forest model differentiating eyes requiring immediate treatment from eyes not requiring treatment demonstrated an AUC of 0.91 ± 0.07. Quantitative angiographic features have potential as important predictive biomarkers of a future need for anti-VEGF therapy in DR and may serve to guide the frequency of a follow-up.

## 1. Introduction

Diabetic macular edema (DME) and proliferative diabetic retinopathy (PDR) are the leading causes of vision loss in diabetic eye disease [1]. High blood glucose levels and advanced glycosylation end products cause vascular damage and result in the upregulation of vascular endothelial growth factor (VEGF) and breakdown of the blood-retinal barrier. This leads to increased retinal vascular permeability and subsequent intraretinal fluid accumulation and macular thickening.

Anti-VEGF injections have become the gold standard therapy for DME, and work by stabilizing damaged blood vessels and promoting fluid reabsorption [2,3,4]. Randomized controlled trials have shown that anti-VEGF therapy can significantly improve macular edema, enhance visual acuity, and reduce diabetic retinopathy severity [5,6]. Recent data has also shown anti-VEGF therapy to be highly effective in regressing retinal neovascularization in PDR [7]. However, predicting who will need anti-VEGF treatment and the timing of the need for intervention remains uncertain when managing diabetic eye disease. This prognostication can play an important role in the frequency and timing of follow-up.

Imaging with optical coherence tomography (OCT) and ultra-widefield fluorescein angiography (UWFA) is critical for the identification and quantification of PDR and macular edema. OCTs provide cross-sectional data, enabling objective monitoring of intraretinal and subretinal fluid in DME. Ultra-widefield imaging has demonstrated its utility to detect more peripheral lesions than the standard 7-field ETDRS imaging [8]. Further, the presence of these peripheral lesions is associated with a 4.7 fold increased risk of progression to proliferative DR [8,9]. Imaging features, such as leakage, microaneurysms, and ischemia on UWFA can aid in determining disease severity, and with emerging technologies can now be objectively quantified [10]. Identifying imaging biomarkers could provide predictive information regarding disease progression and be used in risk stratification for requiring anti-VEGF therapy in the future.

Indications for anti-VEGF therapy in diabetic eye disease include DME, severe nonproliferative DR without DME, and PDR. However, factors that predict future disease progression or the need for anti-VEGF therapy are limited. This study seeks to identify angiographic features in patients with DR that may help to predict future need for anti-VEGF therapy.

## 2. Materials and Methods

An IRB-approved retrospective review of subjects with DR who underwent UWFA using Optos 200Tx or California systems (Optos, Scotland) was performed. The study was approved by the Cleveland Clinical Institutional Review Board, which adhered to the Declaration of Helsinki. Inclusion criteria included the presence of DR and UWFA imaging with at least 1 year (+/− 1 month) of follow-up. Exclusion criteria included prior panretinal laser photocoagulation, intravitreal pharmacotherapy within the last six months, concurrent retinal disease, and poor UWFA image quality [e.g., severe artifacts (e.g., lashes), poor field of view, limited contrast]. The treatment decision was made individually by Cole Eye Institute retina specialists. Clinical features, including age, gender, systolic and diastolic blood pressure, HgA1c at baseline, treatment with anti-VEGF therapy, and follow-up duration were recorded. OCT scans were reviewed for mean central subfield thickness (CST) and the presence of diabetic macular edema (DME).

### 2.1. UWFA Image Analysis

Two images with optimum quality determined by the analyst were selected for each subject, one in the early–mid phase and one in the late phase. Images taken with the Optos 200Tx system were processed by dewarping to correct for peripheral distortion, as previously described [11]. Images from the California system were dewarped through the native software platform.

Ischemia analysis on UWFA was performed through segmentation of areas of capillary nonperfusion in early–mid phase FA images using a previously described image analysis platform [10]. Each image segmentation was reviewed for accuracy by a trained image analyst and sequentially reviewed by an additional expert reader. The total retinal analyzable area was defined as the peripheral edge of the visible retinal vasculature. The segmented areas of ischemia were calculated by summing the area of all pixels. The ischemia index was calculated as the area of ischemia expressed as a percentage out of the total retinal area. Microaneurysm (MA) and leakage segmentation on UWFA were first performed by an automatic feature extraction platform and then reviewed by a trained image analyst, and manually corrected, as needed. A second expert reader sequentially reviewed the final segmentation results for consistency [10,12,13]. MAs were defined as small circular spots of hyperfluorescence compared to surrounding choroidal background in early–mid phase UWFA images. Leakage was defined as a region of increasing hyperfluorescence in size and intensity in the late phase angiogram compared to the early–mid phase FA. Leakage index was calculated as the area of leakage expressed as a percentage out of the total analyzable retina. To assess the regional distribution of MAs and leakage, a mask of three concentric circles of increasing size (macular, midperiphery, and far periphery) was overlaid onto the image and centered around the macula, as previously described. MA count and leakage index were calculated zonally and panretinally [10]. In addition to the zonal indices, distribution of leakage and MA were assessed as the percentage of panretinal leakage and MA count located in the macula and in the periphery.

The retinal vasculature was extracted from the early phase frames using a deep learning algorithm [14,15,16]. Vascular parameters, including panretinal vessel area, length and localized vessel density features were calculated. Localized vessel density was calculated by dividing the vasculature mask into forty by forty pixel squares and measuring the percentage of the areas occupied with retinal vessels in each square. The mean, median, variance, skewness, and kurtosis values of localized retinal vessel density panretinally were included in the analysis.

### 2.2. Statistical Analysis

Eyes were grouped into three categories: (1) eyes not requiring anti-VEGF treatment during the entire follow-up period, (2) eyes requiring anti-VEGF injection within 3 months of the analyzed UWFA, and (3) eyes requiring anti-VEGF after 3 months of an UWFA imaging session. All statistical analyses were conducted using R version 3.6.1 (R Project for Statistical Computing). Statistical analyses were performed for the following clinical variables: age, gender, follow-up period, HbA1c at baseline, systolic and diastolic blood pressure closest to the baseline UWFA date, visual acuity at baseline, DME presence on OCT, and CST. Variables extracted from UWFA images included panretinal, macular, midperipheral and far peripheral leakage index, number of leakage areas and MA count, percentage of leakage and MA distribution in posterior pole, midperiphery and far periphery, panretinal ischemic index, panretinal, macular and midperipheral vessel area and vessel length, vessel area index, mean, median, variance, skewness, and kurtosis values of panretinal localized retinal vessel density and tortuosity. Distribution of normality of the continuous variables was assessed using the Shapiro and Wilk’s normality test. The generalized linear mixed effect model was used to compare each variable individually between the eyes that required anti-VEGF within 3 months, eyes that required anti-VEGF after 3 months, and eyes that did not required any anti-VEGF treatment, while considering intereye correlation. A *p*-value of less than 0.05 was considered statistically significant.

A random forest predictive modeling was used to predict the early and late need for anti-VEGF treatment as their performance is not affected by multicollinearity. Two random forest classifiers using 5 randomly sampled features out of all available imaging and clinical variables (*n* = 45) were used as candidates at each split grown with 1000 trees to differentiate eyes that required anti-VEGF within 3 months (including on the day of UWFA) and those that required it after 3 months of the imaging visit from eyes that did not require anti-VEGF during the follow-up period.

## 3. Results

A total of 173 eyes from patients with diabetic retinopathy were reviewed. The mean follow-up time was 22 (range: 11–43) months. Thirty-eight eyes (22%) required early anti-VEGF treatment within 3 months of the UWFA. Thirty-four eyes (20%) required late anti-VEGF treatment at least 3 months after the UWFA imaging. One hundred and one eyes did not require any anti-VEGF treatment during the follow-up time. There were no significant differences in age, gender, follow-up period, and HbA1c (Table 1).

### 3.1. Early Anti-VEGF Intervention Requirement

**Figure 1:**
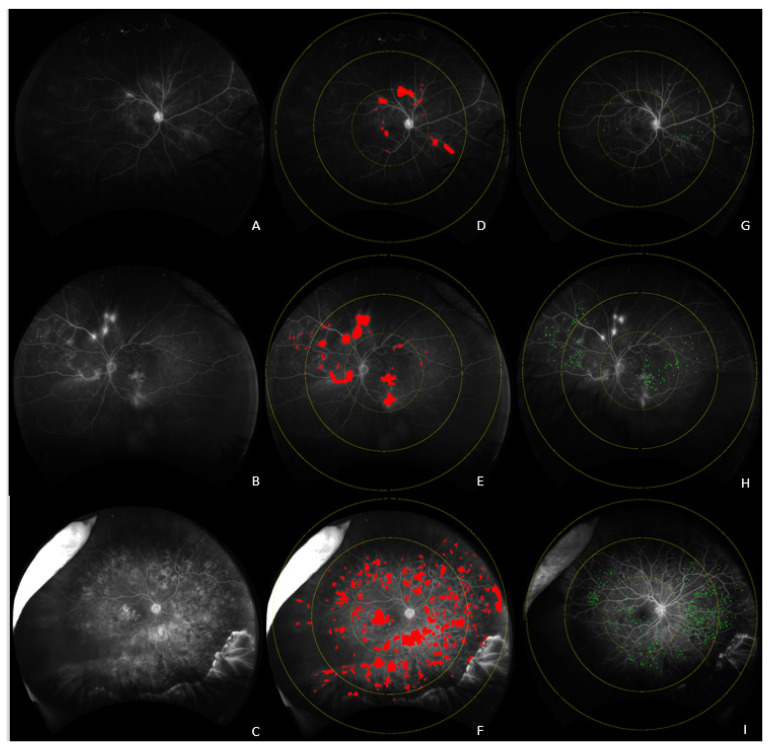
Late UWFA images of eyes not requiring treatment (**A**), requiring delayed treatment (**B**) requiring immediate treatment (**C**), and their corresponding leakage (**D**–**F**), segmentation, and microaneurysm (**G**–**I**) segmentations.

**Figure 2:**
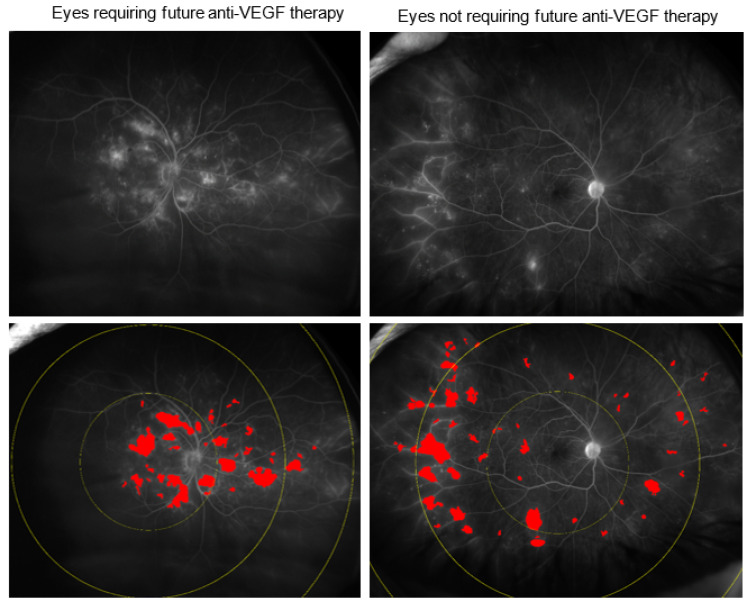
Leakage index in eyes requiring future anti-VEGF therapy. Total leakage index and posterior pole leakage index on UWFA were significantly higher in eyes requiring future anti-VEGF therapy compared to eyes not requiring future injections.

**Figure 3:**
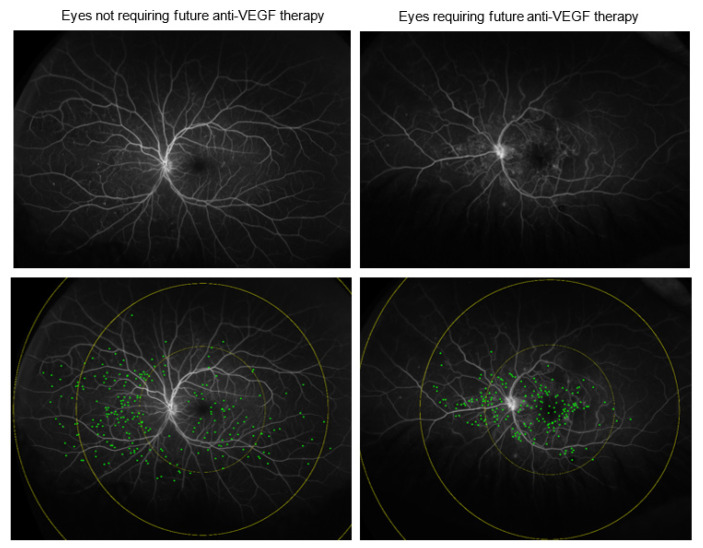
MA count in eyes requiring future anti-VEGF therapy. Total MA count and posterior pole MA count on UWFA were significantly higher in eyes requiring future anti-VEGF therapy compared to eyes not requiring future injections.

At baseline, there were significant differences in multiple imaging biomarkers across the groups. Eyes requiring anti-VEGF treatment within 3 months demonstrated significant differences in the following parameters (Figure 1): panretinal leakage index (4.1% vs. 2.0%, *p* = 0.006), macular leakage index (11.5% vs. 4.3%, *p* = 0.002) (Figure 2), peripheral MA count (167 ± 320 vs. 108 ± 102 *p* = 0.005) (Figure 3), median vessel density (16.6 ± 2.7% vs. 15.1 ± 2.5%, *p* = 0.013), macular vessel area (17.1 ± 2.4 vs. 16.5 ± 2.1 mm^2^, *p* = 0.035), midperipheral vessel area (61.8 ± 16.0 vs. 57.2 ±12 mm^2^, *p* = 0.021), CST (415 ± 134 µm vs. 263 ± 57 µm, *p* = 0.002), DME presence (92% vs. 27%, *p* < 0.001), and visual acuity (20/80 vs. 20/25, *p* = 0.037).

### 3.2. Delayed Anti-VEGF Treatment Requirement

Eyes requiring anti-VEGF treatment after 3 months were also significantly different from eyes not requiring anti-VEGF treatment in the following parameters: macular leakage index (12.9% vs. 4.3%, *p* = 0.007), panretinal leakage index (4.4% vs. 2.0%, *p* = 0.022), visual acuity (20/50 vs. 20/25, *p* < 0.001), and DME presence (65% vs. 27%, *p* < 0.001).

### 3.3. Comparative Assessment of Eyes Requiring Early vs. Deferred Anti-VEGF Treatment

Eyes requiring anti-VEGF treatment within 3 months and after 3 months had significantly different CST (415 ± 134 µm vs. 314 ± 112 µm, *p* = 0.040), DME presence (92% vs. 65%, *p* = 0.011), macular MA count (88 ± 64 vs. 57 ± 46, *p* = 0.044), and macular vessel area (17.1 ± 2.4 vs. 15.5 ± 2.9 mm^2^, *p* = 0.025). This suggests that the primary initial reason for early treatment was the presence/severity of DME.

### 3.4. Automated Classification of Eyes Based on Need for Early or Late Anti-VEGF Therapy

**Figure 4:**
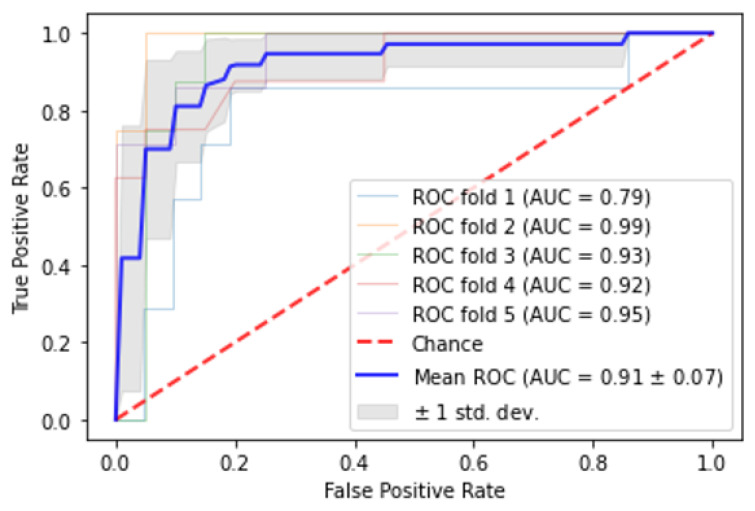
Area under the curve graph for random forest model trained in 5-fold cross-validation setting differentiating eyes requiring immediate anti-VEGF treatment from eyes not requiring treatment.

A random forest model differentiating eyes requiring treatment within 3 months from the eyes not requiring future treatment identified macular leakage index, presence of DME, CST, visual acuity, and macular distribution of leakage as the top five most important features, and demonstrated an AUC of 0.91 ± 0.07 (Figure 4). Random forest model differentiating eyes requiring treatment after at least 3 months from eyes not requiring future treatment demonstrated the top five most important features to be macular leakage index, visual acuity, systolic blood pressure, panretinal leakage index, and follow-up duration and had an AUC of 0.77 ± 0.04.

## 4. Discussion

Anti-VEGF therapy in diabetic eye disease has been shown to improve visual acuity, reduce macular edema, and improve DRSS severity [17,18]. However, it is currently unknown which features predict whether a patient will require anti-VEGF injections. Early stratification of patients at risk for requiring anti-VEGF therapy in the future can help minimize unnecessary office visits and allow initiation of potential interventions sooner to prevent vision-threatening complications of diabetic eye disease.

The present study evaluated the utility of the imaging features in predicting the need for anti-VEGF treatment for the treatment of PDR with or without DME. OCT features including CST and DME presence were significantly different across all groups. This study demonstrated that there is a significant difference in the panretinal and macular leakage index for eyes that required anti-VEGF treatment compared to eyes that did not. These results suggest that the extent of leakage, especially in the posterior pole, may be an important marker for the need for future anti-VEGF treatments, and could potentially be used to modulate follow-up regimens, particularly in eyes without DME. Significantly higher DME presence was observed in eyes requiring immediate treatment compared to delayed treatment, suggesting that the treatment decision was driven by DME. Macular vessel area and macular MA count were also higher in eyes requiring immediate VEGF treatment compared to delayed treatment. The associations between the disease progression to treatment threshold and the angiographic features including macular vessel area and MA count should be further investigated.

Along with the intraretinal microvascular abnormalities, MAs are among the vascular abnormalities associated with DR severity. The number and turnover rate of MAs have previously been considered an important biomarker for the progression of DME [19]. The findings in this analysis indicate that MA count is associated with requiring anti-VEGF therapy. MA count in the posterior pole and macular vessel length were both significantly higher in eyes that required treatment within 3 months. MAs are hypothesized to be focal areas of permeability in the retinal vasculature that may give rise to leakage [20]. Anti-VEGF treatments for DME have been shown to decrease diffuse leakage but have relatively little effect on focal leakage as assessed by FA [21]. Another study found a significant decrease in microaneurysms after anti-VEGF therapy [22]. The relationship between anti-VEGF therapy and MA count requires additional investigation.

Larger vessel area measured in the macular zones of eyes requiring immediate treatment may be due to active vascular remodeling. In a recent study, increased macular vessel area was extracted from the UWFA frames of moderate and severe NPDR eyes compared to PDR eyes [23]. Another study demonstrated a higher risk of DME development in moderate and severe NPDR compared to PDR [24]. Further studies investigating the integrative impact of DR severity compared or combined with quantitative angiographic features on future treatment should be explored. The current study also demonstrated higher panretinal vascular density measures in eyes requiring immediate anti-VEGF treatment. Based on current vascular extraction platforms, increased vascular remodeling in the setting of increased disease activity often leads to greater contrast and enhanced visualization of individual vessels and may account for the higher vessel density measurements.

The present study did not demonstrate a difference in ischemia on UWFA in eyes with DR with different anti-VEGF needs. Previous studies have found variable results on the association of peripheral retinal ischemia with the presence of DME [9,25,26]. UWFA images from eyes with DR before and after anti-VEGF injections suggest that areas of ischemia do not demonstrate significant reperfusion following anti-VEGF therapy despite an improvement in the DRSS score based on color fundus photographs [7,12,17,27]. The findings from our study suggest that ischemia may not be a key predictor for the anti-VEGF therapy requirement in DR; but rather that the leakage index may reflect more current vascular disease activity and may be more indicative of a future need for anti-VEGF therapy, whereas ischemia may be more reflective of overall disease effects and not current disease activity.

Random forest analysis demonstrated that macular leakage index, visual acuity, CST, and DME were the most important features differentiating the three groups. The most successful model differentiating eyes requiring anti-VEGF treatment in the near future from eyes not requiring treatment demonstrated an AUC of 0.91. The utility of these models as clinical decision-making tools should be further investigated, particularly in relation to follow-up time and risk stratification.

The strengths of this study include the use of UWFA to capture both peripheral and nonperipheral angiographic features implicated in DR. The panretinal leakage index and peripheral MA count, which could not be visualized by standard FA, were significantly higher in eyes that required anti-VEGF treatment within 3 months. Moreover, the panretinal leakage index was an important variable predicting delayed anti-VEGF treatment. Semiautomated quantitative analysis of these imaging features provides a novel way to assess the extent of leakage, MAs, ischemia and vascular features on UWFA with improved objectivity. Several studies have reported therapeutic effects in the fellow eye following unilateral anti-VEGF injections [28,29,30]. For subjects that had two eyes included in the study, a linear mixed effects model was used to correct for dependence. Random forest predictive modeling was used to predict the early or delayed anti-VEGF needs for its ability to handle complex dependency patterns between correlated covariates. A limitation of this study included that the software used to segment areas of ischemia was unable to provide detailed analysis into the distribution of ischemia regions (e.g., in the posterior pole). Future studies should explore the regional differences in ischemia in DR. Additionally, DM duration, pharmacotherapy 6 months prior to the baseline visit, and axial length measurements of the patients were not available. Pixel to mm^2^ conversions were not adjusted to actual axial length. Another limitation of this study was a relatively small sample size when stratifying the timing of future anti-VEGF injections. A larger sample size would allow further delineation of the temporal relation between angiographic imaging features and future therapies in DR. Lastly, there were no preset indications guiding the treatment decision in this retrospective study, and retina specialists individually determined the appropriate treatment for each patient. This study identified quantitative imaging features on UWFA that predict the need for future anti-VEGF therapy; however, it was not possible to delineate the clinical indication for the injection (e.g., DME, DR, or both). Future studies should also investigate whether specific imaging features are predictive of the development of DME, PDR, or both.

In conclusion, quantitative leakage on UWFA along with OCT features are useful for identifying eyes with DR that likely need future anti-VEGF therapy. Additional research is needed to determine the underlying mechanisms allowing the angiographic prediction of intravitreal treatment for DR and treatment response.

## Figures and Tables

**Table 1:** Summary of select characteristics and imaging features for each treatment group.

	Not Requiring Treatment (*n* = 101)	Anti-VEGF Injection within 3 Months (*n* = 38)	Anti-VEGF Injection after 3 Months (*n* = 34)
Age	63 ± 13	66 ± 13	62 ± 13
Gender			
Female	48	20	17
Male	53	18	17
Follow-up time (months)	21.6 ± 8.3	21.0 ± 10.3	25.7 ± 10.8
Systolic Blood Pressure	134.6 ± 15.6	139.5 ± 19.8	144.6 ± 20.2
Hypertension	97.0%	100%	97.1%
Presence of DME on OCT *	27%	92%	65%
HbA1c	8.0 ± 1.8	8.4 ± 2.3	8.4 ± 2.3
Visual acuity *	20/25	20/80	20/50
CST (µm) *	263 ± 57	415 ± 134	314 ± 112
Panretinal LI (%) *	2.0 ± 2.8	4.1 ± 3.4	4.4 ± 3.7
Macular LI (%) *	4.3 ± 5.0	11.5 ± 7.6	12.9 ± 11.3
Panretinal ischemia index (%)	2.1 ± 1.4	3.1 ± 4.1	4.7 ± 4.8
Panretinal MA count	161 ± 131	260 ± 385	156 ± 125
Macular MA count *	51 ± 46	88 ± 64	57 ± 46
Peripheral MA Count *	108 ± 102	167 ± 320	97 ± 93
Panretinal vessel area (mm^2^)	86.2 ± 19.8	92.2 ± 25.3	90.6 ± 25.0
Macular vessel area (mm^2^) *	16.5 ± 2.1	17.1 ± 2.4	15.5 ± 2.9
Median vessel density (%) *	15.1 ± 2.5	16.6 ± 2.7	16.1 ± 2.6

Abbreviations: CST: central subfield thickness, DME: diabetic macular edema, LI: leakage index, MA: microaneurysm. * significantly different among categories.

## Data Availability

Additional data available upon request.
